# Frequent drinking of small volumes improves cardiac function and survival in rats with chronic heart failure

**DOI:** 10.14814/phy2.13497

**Published:** 2017-11-09

**Authors:** Can Zheng, Meihua Li, Toru Kawada, Masashi Inagaki, Kazunori Uemura, Masaru Sugimachi

**Affiliations:** ^1^ Department of Cardiovascular Dynamics National Cerebral and Cardiovascular Center Osaka Japan

**Keywords:** Chronic heart failure, drinking behavior, fluid restriction, survival, thirst

## Abstract

Fluid retention is the main reason for the high hospitalization rate among patients with chronic heart failure (CHF). Given the lack of knowledge about fluid intake regulation and its consequences in patients with CHF, current guidelines do not provide clear direction for fluid management. Using a rat model of CHF, we investigated altering drinking behaviors and explored fluid management strategies. CHF was induced by ligating the left anterior descending coronary arteries in 8‐week‐old, male, Sprague‐Dawley rats. A custom‐designed drop counting and feedback control system was used to record and modulate drinking behaviors. During the first month after an induced myocardial infarction (MI), we observed that the spontaneous per drinking volume (PDV) was significantly increased in animals with prolonged intervals between drinking episodes. In addition, there was a significant inverse correlation between the early PDV and the post‐MI lifespan (*r* = −0.907; *P* < 0.001). Moreover, modulating the drinking behavior of rats with CHF to involve frequent drinking of small PDVs significantly enhanced hemodynamics and prevented cardiac remodeling, with a significant improvement in the 180‐day survival rate, compared with animals allowed to drink freely (50% vs. 36%; *P* < 0.01). The results of dynamic PDV changes, after MI, suggest that an impaired thirst mechanism is associated with the sensing and regulating of fluid balance in rats with CHF. These results suggest that increasing the drinking frequency, with small PDVs, may be beneficial to preventing progression of cardiac dysfunction in CHF.

## Introduction

Although major advances have been made in the treatment of chronic heart failure (CHF), it remains a major global public health issue, with a high prevalence, poor clinical outcome, and large burden of hospitalization costs.(Desai and Stevenson 2012) The admission rates for acute decompensated heart failure remain high, with a readmission risk of approximately 25% within 30 days and >50% within 180 days.(Ross et al. 2010) Therefore, improving the self‐care ability of patients is a novel potential strategy for reducing readmissions.(Riegel et al. 2009)

In outpatients with CHF, recurrent congestion—usually due to fluid retention—is the most frequent cause of re‐hospitalization. Fluid retention is caused by an imbalance in fluid intake and output. Currently available therapy, which mainly focuses on increasing urinary output via pharmaceutical diuretics, often fails to prevent recurrent congestion after discharge (Lala et al. 2015). Recently, fluid management in patients with CHF has been a matter of concern,(Holst et al. 2008) and several clinical trials have evaluated the consequences of fluid and salt restrictions (Li et al. 2015). However, due to the small sample sizes and heterogeneity of the patients' conditions in these studies, and no basic knowledge support daily fluid restriction, the conclusions regarding the potential benefit of fluid restriction remain unclear. Moreover, the current guidelines for the management of heart failure provide only vague and generic advice about fluid restriction (Yancy et al. 2013).

As the clinical use of various therapeutic interventions clouds our understanding of the natural pathophysiological processes, there is inadequate knowledge about the pathophysiological changes associated with fluid regulation in CHF (Philipson et al. 2010, 2013). Recent studies have indicated that thirst increases and persists in patients with CHF (Waldreus et al. 2013, 2014). In particular, patients who adhere to daily fluid restriction recommendations often experience increased thirst (Reilly et al. 2015). Thirst is a sensation and desire that elicits drinking behavior, and is usually evaluated in patients using a visual analog scale (VAS) (Allida et al. 2014). However, as VAS evaluations are subjective and cannot be continuously recorded, data regarding the development of thirst and the effects of its management are scarce in both clinical practice and animal models of CHF.

In this study, we investigated drinking behavior using a custom‐designed drop counting system with normal and CHF rats. The most notable change was that the spontaneous per drinking volume (PDV) markedly increased after an induced myocardial infarction (MI), and that this increase was sustained throughout the rat's lifespan. We deduced that consuming fluid with a large PDV and a longer between‐drink interval might increase the disturbance of the internal environment, potentially leading to the progression of cardiac dysfunction. To verify this hypothesis, we modulated the drinking behavior of CHF rats, having induced MI, to reduce the PDV and shorten the between‐drink interval. The effects of PDV restriction on cardiac remodeling and survival were then evaluated. As a comparison, we reproduced the daily fluid restriction that is recommended in clinical trials and verified the behavioral and hemodynamic responses in this model.

## Methods

### Animal model of CHF

The care and use of animals conformed to the recommendations in the Guide for the Care and Use of Laboratory Animals (US National Institutes of Health, Bethesda, MD) and the Guiding Principles for the Care and Use of Animals in the Field of Physiological Sciences (Physiological Society of Japan, Tokyo). All protocols were reviewed and approved by the Animal Subject Committee of the National Cerebral and Cardiovascular Center. The animals had free access to food (CLEA Rodent Diet CE‐2, CLEA Japan, Inc.), which contained the following main minerals: Na (0.31%), K (1.02%), Ca (1.06%), and Mg (0.34%). The animals were individually housed in standard cages in a temperature‐controlled room (25 ± 2°C), which was illuminated from 6:00 AM to 6:00 PM. The body weights of the animals were measured weekly. The animals were examined daily to assess their health and to record any deaths.

**Figure 1:**
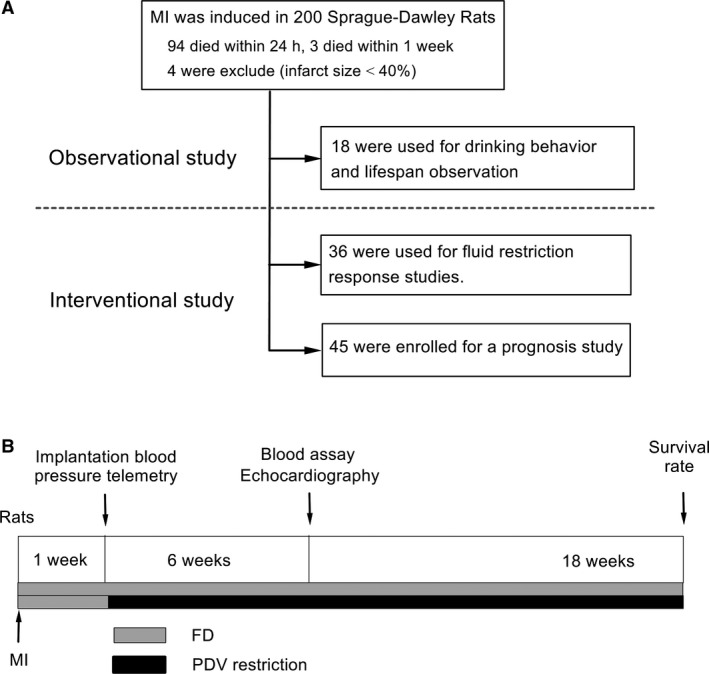
Schematic description of the study design and timeline. (A) Study design for drinking behavior observations and modification in rats with chronic heart failure (CHF). The surviving CHF rats were randomly assigned to receive free drinking (FD) or per drinking volume (PDV) restriction programs for hemodynamic response, remodeling, and prognosis studies. (B) Study events and timeline. MI, myocardial infarction.

Left ventricular MI were induced, via coronary artery ligation, in 8‐week‐old, male, Sprague‐Dawley rats (*n* = 214; Japan SLC, Inc.) under halothane inhalation anesthesia (3% at introduction and 1.2% during surgery). The mortality rate in the animals with MI was approximately 50% (*n* = 101) within the first 24 h, and an additional three rats died within the first week. The survivors (*n* = 110) were enrolled in this study (Fig. 1). We examined the postmortem infarct size in the rats, (Li et al. 2014) and those with an infarct area <40% of the left ventricular wall were excluded from the study (*n* = 4). In addition, normal rats (*n* = 10) were used to study drinking behaviors and to develop the flow control system. At the end of the experiment, rats were sacrificed under deep halothane (5%) anesthesia.

### Modulation of drinking behavior and fluid restriction interventions

We developed an automatic flow control system for small animals (Zheng et al. 2013). In brief, this system consists of a drop sensor for measuring fluid flow and an electric valve for feedback control of the flow. A software program counts the drops and precisely controls the flow; when the counts reach a preset value, the program shuts the valve and opens it after a preset interval. To help the animals adapt to the control system, an LED indicator, adjacent to the nozzle, is synchronized with the valve state to establish a conditioned reflex.

With this system, we evaluated two types of fluid restriction programs: per drinking volume (PDV) restriction and daily drinking volume (DDV) restriction. For PDV restriction, the valve was opened and counting was initiated at 6:00 PM. Thereafter, the PDV was individualized according to Formula 1. When the counts reached the preset PDV, the valve was shut and re‐opened after a 30‐min interval. For DDV restriction, the valve was opened and counting was initiated at 6:00 pm. If the total daily fluid consumption reached the target DDV, the valve was shut until the subsequent 6:00 pm.

(1) $$\begin{array}{cc} & \text{Per drinking volume}=\\ & \frac{\left(\text{Targeted daily volume})\times (\text{Body weight}\right)}{\left(\text{Targeted drinking frequency}\right)} \end{array}$$

The timings of each drop and the valve control command were recorded in a laboratory personal computer for off‐line analysis.

### Telemetry device implantation

To examine hemodynamic responses to drinking behavior modulation, at 1 week post‐MI induction, rats (*n* = 12) received implanted blood pressure telemetry devices (TA11PA‐C40; Data Science International, St. Paul, MN) under halothane anesthesia (3% for intubation and 1.2% during the surgery). The tip of the sensor was inserted into the abdominal aorta, and the transmitter was sutured to the abdominal wall. The mean arterial pressure (MAP) and heart rate (HR) data were recorded continuously in the non‐stressed animals. The recording was sampled at 500 Hz. Tests for the hemodynamic responses to fluid restriction (PDV restriction or DDV restriction) were conducted 2 weeks after telemetry device implantation.

### Echocardiography

To evaluate cardiac remodeling and dysfunction in the PDV restriction (*n* = 5) and the free drinking (FD, *n* = 7) groups after 7 weeks of treatment, echocardiographic studies were conducted using an echocardiography system equipped with an MS‐250 transducer (Vevo^®^ 2100 system, VisualSonics, Toronto, ON, Canada) under isoflurane anesthesia (1.0%).

### Neurohumoral and biochemical study

Blood sampling was performed in anesthetized (1.2% halothane) animals after 7 weeks of FD (*n* = 6) or PDV restriction (*n* = 6). Blood samples were collected from the right jugular veins, and the recovered plasma and serum samples were maintained at −80°C for later analyses. Plasma norepinephrine concentrations were measured using high‐performance liquid chromatography with electrochemical detection. The plasma levels of brain natriuretic peptide (BNP; BNP‐32 Enzyme Immunoassay Kit, Peninsula Laboratories. San Carlos, CA) and arginine vasopressin (AVP, arg8‐Vasopressin Enzyme Immunoassay Kit, Assay Designs, Ann Arbor, MI) were determined using enzyme‐linked immunosorbent assays. The levels of serum sodium and potassium, and other factors associated with kidney and hepatic function were measured using an automatic analyzer (model 7020, Hitachi, Tokyo, Japan).

### Long‐term survival study

To examine the effect of PDV restriction on prognosis, we assessed the 180‐day survival rate among CHF rats in the PDV restriction (*n* = 20) and FD (*n* = 25) groups. PDV restriction was initiated 1 week after MI induction and continued for 180 days.

### Statistical analysis

All data are expressed as means ± standard errors. The data recorded during the behavior and hemodynamic studies were evaluated using Student's *t*‐test. Linear regression analysis was used to explore the relationship between fluid consumption and post‐MI lifespan. The Mann–Whitney *U*‐test was used to detect neurohumoral or biochemical differences between the groups. The survival data are presented as Kaplan–Meier curves, and the effect of PDV restriction on 180‐day survival was analyzed using a log‐rank (Mantel‐Cox) test. All differences were considered to be significant at a *P* < 0.05.

## Results

### Pathological drinking behavior changes after MI

**Figure 2:**
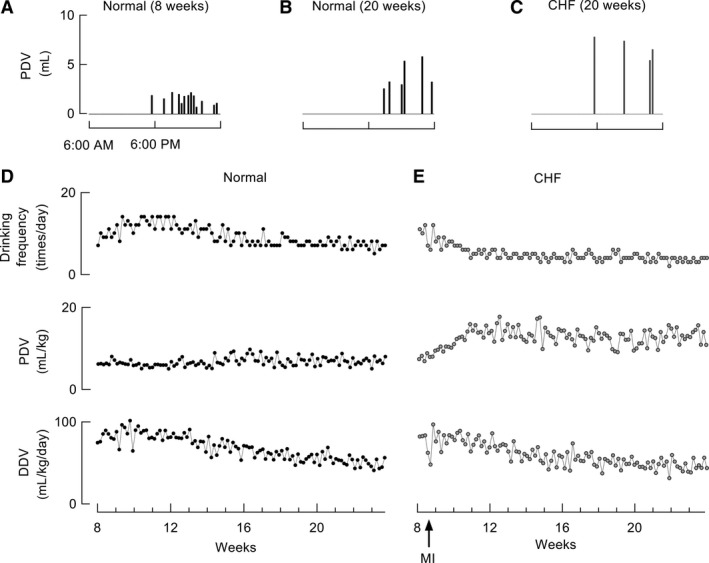
Fluid consumption and drinking behavior in normal rats and those with chronic heart failure (CHF). (A–C) Representative 24‐h drinking behavior in normal (8 and 20‐week‐old) and CHF (20‐week‐old) rats. D and E, Typical long‐term profile of drinking behavior in normal and CHF rats.

**Figure 3:**
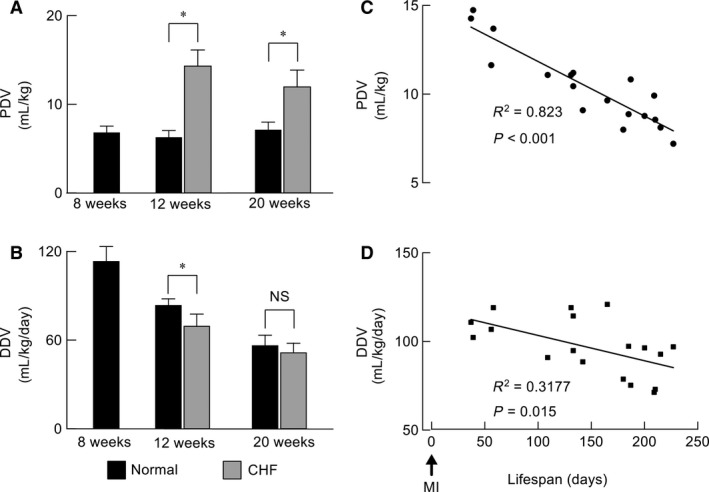
Drinking behavior and its relationship with lifespan in rats with chronic heart failure (CHF). (A and B) Comparison of per drinking volume (PDV) and daily drinking volume (DDV) in normal (*n* = 6) and CHF (*n* = 6) rats. (C and D) The relationship between early (second week after myocardial infarction) daily drinking volume (DDV) or per drinking volume (PDV) and the lifespan of CHF rats (*n* = 18). Data are presented as means ± standard deviations and were compared using Student's t‐test. *P < 0.01; NS, not significant. The lifespan was considered as the period from the day of myocardial infarction induction. MI, myocardial infarction.

As rats are nocturnal in nature, they consume most of their food and water during the night, and rest or sleep during the day. Normal, young (8‐week‐old) rats were observed to drink more than 10 times/day, with small PDVs (Fig. 2A). Although the drinking frequency decreased and the absolute PDV gradually increased with age (Fig. 2B), the PDV, adjusted for body weight, remained unchanged, and was relatively low (Fig. 2D). After an induced MI, the most distinct change was an increase in PDV during the initial 1–4 weeks, both in terms of the absolute value and the body weight‐adjusted value; this increase was sustained at a higher level than in normal rats throughout the remaining lifespan (Figs. 2C, E, and 3A). The body weight‐adjusted DDV decreased with age in both normal and CHF rats (Fig. 2D and E); there was no significant difference in the body weight‐normalized DDV between normal and CHF animals at 20 weeks of age (Fig. 3B).

### Correlation between PDV/DDV and post‐MI lifespan

To explore the relationship between fluid consumption and prognosis, we assessed drinking behavior shortly (second week) after the induced MI and the post‐MI lifespan. As shown in Figure 3, unlike the weak correlation with DDV, PDV was strongly correlated with the post‐MI lifespan (Figs 3C and D).

### Effects of PDV restriction on CHF

#### Daily fluid consumption

**Figure 4:**
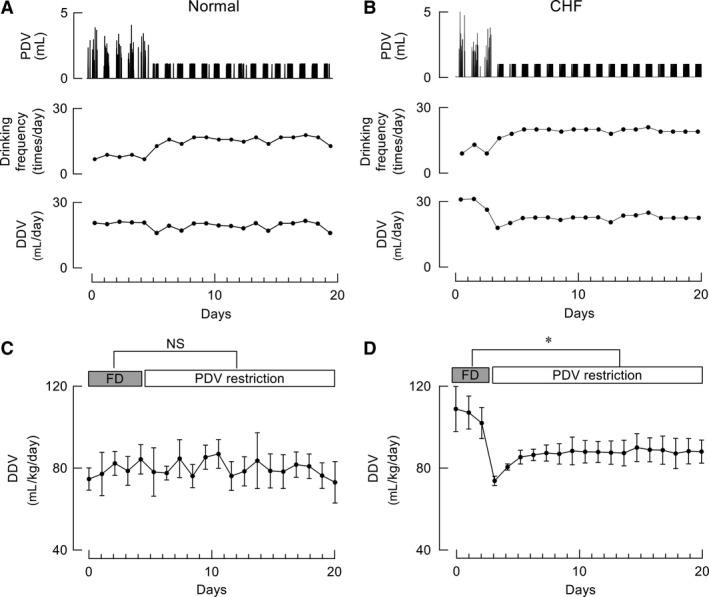
Effects of per drinking volume (PDV) restriction on daily fluid consumption in normal rats and in those with chronic heart failure (CHF). (A) Representative responses to PDV restriction in normal (12‐week‐old) rats. (B) Representative responses to PDV restriction in CHF (10‐week‐old) rats. (C and D) Averaged daily drinking volume (DDV) before and after PDV restriction in adult normal (*n* = 6) and CHF (*n* = 7) rats. Student's *t*‐test. * *P* < 0.01; NS, not significant; FD, free drinking.

The daily fluid consumption responses to PDV restriction differed between the normal and CHF rats. In the absence of daily limitations, normal rats showed almost no change in DDV (Fig. 4A and C) before and after PDV restriction. In contrast, CHF rats exhibited a significantly reduced DDV during PDV restriction (Figs. 4B and D).

#### Hemodynamic responses

**Figure 5:**
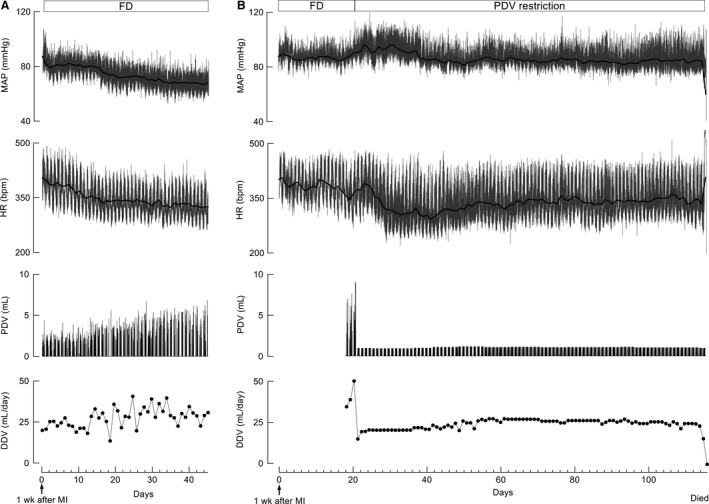
Drinking behavior and hemodynamic responses to free drinking (FD) and per drinking volume (PDV) restriction in rats with chronic heart failure (CHF). (A) Typical natural drinking behavior and hemodynamic changes in a FD CHF rat. (B) Typical hemodynamic responses to PDV restriction in CHF rats. Daily fluid consumption decreased when treated with PDV restriction, mean arterial pressure (MAP) increased and then remained relatively stable level during the treatment. The resting phase heart rate (HR) decreased while the active phase HR remained high. The cause of death was sustained tachyarrhythmia leading to pump failure.

**Figure 6:**
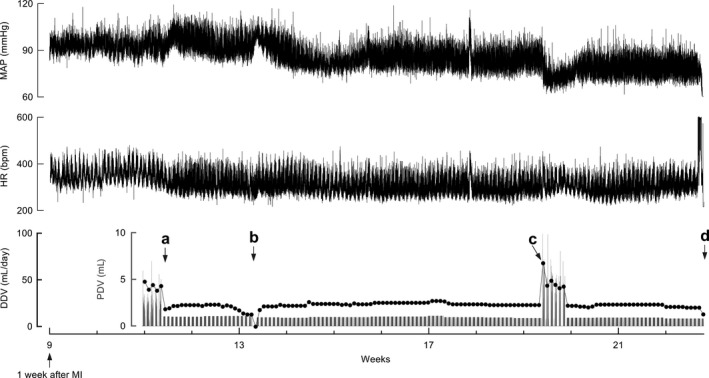
Hemodynamic responses to drinking behavior changes in a rat with chronic heart failure (CHF), determined via telemetry. (A) Initiation of per drinking volume (PDV) restriction. The mean arterial pressure (MAP) was increased, whereas the heart rate (HR) decreased in response to PDV restriction initiated at 2 weeks after myocardial infarction. (B) Decreased drinking volume caused by flow tract obstruction. (C) Discontinuation of the restriction program led to free drinking. Both PDV and DDV markedly increased. The MAP decreased to the lowest value while HR increased. (D) Death due to sustained tachyarrhythmia. MI, myocardial infarction.

In this study, a telemetry system was used to monitor non‐stress hemodynamics and evaluate responses to fluid restriction. Among FD CHF rats, the MAP decreased to 70 mmHg within 1 month after MI (Fig. 5A). We compared the data before (3 days) and after (3 days from the second day) PDV restriction. As exemplified in Figures 5B and 6A, MAP increased significantly (from 88.6 ± 4.3 mmHg to 93.7 ± 4.4 mmHg, *n* = 5; *P* < 0.05), whereas HR decreased (from 360 ± 19 bpm to 319 ± 7 bpm, *n* = 5; *P* < 0.05). These improvements were observed 1–2 days after the PDV restriction program was initiated, and MAP remained >80 mmHg during following stable phase. In contrast, when the PDV restriction program was switched to FD, the PDV and DDV of CHF rats increased, leading to decreased MAP and increased HR (Fig. 6C).

#### Echocardiography, neurohumoral tests, and blood assays

**Figure 7:**
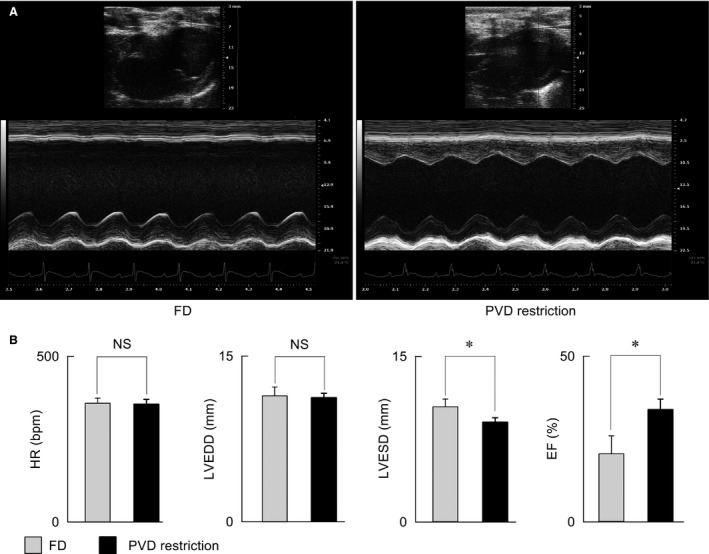
The effect of per drinking volume (PDV) restriction on left ventricular remodeling and dysfunction, as assessed using echocardiography. (A) Representative M‐mode echocardiograms from the free drinking (FD) and PDV restriction groups. (B) Measurements of heart rate (HR) and left ventricular dimension (FD: gray bars, *n* = 7; PDV restriction: black bars, *n* = 5). LVEDD, left ventricular end diastolic diameter; LVESD, left ventricular end systolic diameter; and EF, ejection fraction. Data are presented as means ± standard deviations, and were compared using Student's *t*‐test. *P < 0.01; NS, not significant.

Echocardiography measurements were performed on anesthetized CHF rats after 7 weeks of PDV restriction. The ejection fraction (EF) was higher in the PDV restriction group than in the FD group mainly due to smaller left ventricle end systolic diameters (Fig. 7). Furthermore, blood samples were collected from anesthetized animals after 7 weeks of PDV restriction, and compared with those from the FD group (Table 1). The plasma BNP, AVP, and norepinephrine levels were lower in the PDV restriction group than in the FD group. In addition, albumin and high‐density lipoprotein cholesterol levels were higher, whereas creatinine levels were lower, in the PDV restriction group, than in the FD group.

**Table 1:** Results of neurohumoral and biochemical assays of blood samples of rats with chronic heart failure in the free drinking (FD) and per drinking volume (PDV) restricted groups

	FD group (*n* = 6)	PDV restriction group (*n* = 6)	*P* value
Plasma			
BNP, pg/mL	453 ± 57	382 ± 9	0.01
AVP, pg/mL	676 ± 129	424 ± 204	0.03
NE, pg/mL	815 ± 236	521 ± 155	0.03
Serum			
Total protein, g/dL	5.8 ± 0.4	6.2 ± 0.2	NS
Albumin, g/dL	1.9 ± 0.2	2.2 ± 0.1	0.01
Creatinine, mg/dL	0.35 ± 0.06	0.24 ± 0.03	0.01
BUN, mg/dL	21.4 ± 2.5	20.6 ± 4.1	NS
Na, mEq/L	139.4 ± 2.7	139.2 ± 1.3	NS
K, mEq/L	6.7 ± 0.8	5.8 ± 1.2	NS
Glu, mg/dL	206 ± 24	232 ± 29	NS
HDL, mg/dL	17.6 ± 1.5	24.2 ± 4.6	0.01

Data are expressed as means ± SD. *P* value, significance of difference between the FD and PDV restriction groups, assessed using the Mann–Whitney *U*‐test. BNP, brain natriuretic peptide; AVP, arginine vasopressin; NE, norepinephrine; BUN, blood urea nitrogen; Glu, Glucose; HDL, high‐density lipoprotein cholesterol; NS, not significant.

#### Long‐term survival

**Figure 8:**
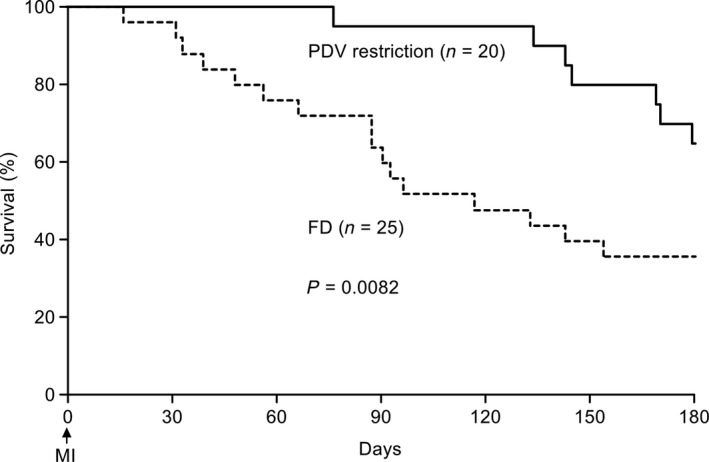
Survival, analyzed using Kaplan–Meier curves, from the induction of myocardial function (MI) in free drinking (FD, dotted line, *n* = 25) and per drinking volume (PDV) restriction (solid line, *n* = 20) rats. PDV restriction was initiated 1 week after coronary artery ligation, and significantly improved the survival rate (*P* = 0.0031, log‐rank test). MI, myocardial infarction.

As observed in the Kaplan–Meier 180‐day survival curves (Fig. 8), the survival rate in the PDV restriction group (*n* = 20) was significantly higher than in the FD group (*n* = 25). In particular, the 180‐day survival rate was significantly higher in the PDV restriction group than in the FD group (50% vs. 36%, *P* < 0.01). The median survival period was 116 days in the FD group and >180 days in the PDV restriction group. There were no significant differences between the FD and PDV restriction groups in postmortem body weight (488 ± 111.1 g vs. 519 ± 107.5 g, *P* > 0.05), bi‐ventricle weight (3.43 ± 0.84 g/kg vs. 3.28 ± 0.64 g/kg, *P* > 0.05), or myocardial infarction size (46.7 ± 3.3% vs. 47.1 ± 3.8%, *P* > 0.05).

### Effects of DDV restriction on drinking behavior and hemodynamics

**Figure 9:**
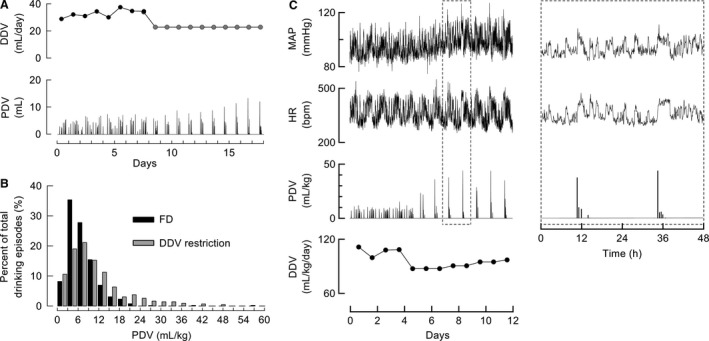
Effects of daily drinking volume (DDV) restriction on drinking behavior and hemodynamics, determined via telemetry, in rats with chronic heart failure (CHF). (A) Typical drinking behavior response to DDV restriction in a CHF rat. (B) Distribution of PDV in CHF rats with free drinking (FD) and DDV restriction. Black bars represent FD (*n* = 4, 648 traces), whereas gray bars represent DDV restriction (*n* = 4, 426 traces). (C) Representative hemodynamic responses to DDV restriction: the mean arterial pressure (MAP) increased, but heart rate (HR) did not decrease. (D) Enlargement of the dotted line frame in C to show the detailed MAP and HR response to DDV restriction.

We also assessed drinking behaviors and hemodynamic responses to DDV restriction in CHF rats. In the DDV restriction study, the maximum DDV was limited to 75% of the baseline DDV. This rate (75%) was selected based on the responses to DDV and PDV restriction in CHF rats. When DDV restriction treatment was initiated, the average PDV gradually increased to a level that was twice as high as the baseline PDV (Fig. 9A). A PDV histogram indicated that the frequency of large PDVs increased after DDV restriction (Fig. 9B). Although the MAP increased after DDV restriction, the average HR did not decrease (Fig. 9C). The sudden ingestion of a large volume after DDV restriction led to several hours of sustained high HR and high MAP in CHF rats (Figs. 9C and D).

## Discussion

To the best of our knowledge, this is the first study to examine post‐MI drinking behaviors and evaluate long‐term outcomes of fluid restriction in a rat model of CHF. In this study, we observed that increases in PDV occurred at an early stage after MI induction and the increase continued during the progression of cardiac dysfunction; daily body weight‐adjusted fluid consumption, in stable CHF animals, was similar to that in normal rats; the modulation of drinking behavior in CHF animals to include small PDVs and short drinking intervals significantly improved cardiac function, reduced plasma BNP, AVP, and norepinephrine levels, and improved long‐term survival.

Drinking behavior is controlled by a thirst mechanism that regulates the body's fluid balance. Impairment of the thirst function may lead to a fluid imbalance that is harmful to homeostasis. In recent clinical trials, a VAS method indicated that thirst is increased in CHF patients (Waldreus et al. 2011, 2014); however, this measurement only serves as a qualitative index related to quality of life.(Reilly et al. 2010) A quantitative measurement of thirst is essential for understanding and treating fluid disorders related to disease, such as fluid retention in CHF.

Because the thirst mechanism serves to control the timing and volume of each drinking episode, the measurement of spontaneous PDV in a no‐stress environment is reasonable for quantitatively evaluating thirst. By using a drop sensor, the PDV—used as an index of thirst in this study—can be continuously monitored in FD rats. Thus, we observed this natural process in an animal model of CHF, in the absence of any therapeutic interference; such observations are almost impossible in clinical situations. One of our main findings was that early PDV increases closely correlate with a short post‐MI lifespan. Thus, increased PDV appears to be an important factor in influencing the progression of cardiac dysfunction. During the stable stage of CHF, we noted that animals drank as much as 20 mL/kg during a single drinking episode. If we extrapolate this dose to humans, an individual weighing 50 kg would be expected to drink 1000 mL at one time. Drinking such a large volume to satisfy one's thirst would be unlikely, without recognizing the adverse consequences of acute volume loading.

The DDV during the stable stage of CHF was similar to that in normal rats, suggesting that the basal fluid requirement in CHF patients is related to the normal consumption in healthy individuals. Hence, the aggressive restriction of DDV to a limit that is lower than that of a healthy individual would be inappropriate, and may only increase the level of thirst without any expected benefit (Aliti et al. 2013). However, during DDV restriction intervention, we observed that the PDV increased significantly over a short time period, providing quantitative evidence that limiting daily consumption may lead to increased thirst and the risk of acute volume loading in CHF.

Our feedback control system that modulates animal drinking behavior is a methodological innovation. The successful introduction of the conditioned reflex may have minimized any potential fluid restriction‐related stress in the experimental animals. Behavior studies have shown that animals can adapt to PDV restriction programs within 1–2 days, which is expressed via shortening of the response time to the conditional signal and a decrease in futile licking activity. This enables long‐term fluid restriction treatment and appropriate outcome evaluation (Zheng et al. 2013).

To our knowledge, this is the first study to control PDV in the CHF disease model. Normal rats did not exhibit major changes in their DDV during PDV restriction. However, the DDV decreased in CHF rats (Figs. 4C and D). The mechanisms underlying these different responses to PDV restriction remain unclear.

In this study, we used a low‐stress telemetry system to monitor hemodynamic responses to fluid restriction. Drinking behavior exerts a major impact on the hemodynamics of CHF rats. In particular, PDV restriction significantly improved hemodynamics by increasing the MAP and reducing the HR within 1–2 days, and these effects were maintained during the treatment period. We could easily reverse or repeat the PDV restriction effects by simply initiating or discontinuing the treatment program (Fig. 6). Echocardiography data indicated that hemodynamic improvements in the PDV restriction group, relative to the FD group, are associated with enhanced cardiac function. In addition, CHF rats experiencing PDV restriction showed lower plasma creatinine levels than did those in the FD group, indicative of better renal function. Moreover, the plasma catecholamine and BNP level reductions s were consistent with the abovementioned beneficial effects of PDV restriction treatment (Table 1).

Overall, compared with CHF rats in the FD group, simply modulating the drinking behavior to include small PDVs and short drinking intervals effectively improved the hemodynamics and prevented the progression of cardiac dysfunction. These benefits were ultimately associated with improved long‐term survival in CHF rats.

In this study, we considered that increased PDV may be a therapeutic target, and confirmed the effectiveness of PDV restriction treatment in CHF rats. At least two mechanisms may be proposed to explain the treatment effects of PDV restriction. First, increased PDV combined with long drinking intervals in CHF may cause increased disturbance of the circulatory system. Acute ingestion of a large volume will increase the burden on a weakened heart, and long intervals between drinks may lead to dehydration that may promote compensatory sympathetic activation. Hence, a small PDV may reduce the risk of acute fluid overload. Moreover, short between‐drink intervals may prevent dehydration and exert beneficial effects for maintaining fluid homeostasis. The results of our telemetric MAP and HR measurements, which remained relatively stable during PDV restriction treatment, may support this mechanism.

Second, AVP is reportedly upregulated and exhibits a defective response to physiological stimulation in CHF (Goldsmith et al. 1983; Goldsmith 1992). Clinical trials proved that AVP antagonism yields acute benefits in acute decompensated heart failure (Gilotra and Russell 2014). Drinking behavior is known to effectively suppress AVP secretion via oropharyngeal stimulation (Geelen et al. 1984; Salata et al. 1987; Thrasher et al. 1987; Figaro and Mack 1997). We hypothesize that increasing the drinking frequency may suppress plasma AVP levels, in turn increasing renal urine production and suppressing thirst. The lower plasma AVP level in the PDV restriction group, compared with the FD group, supports our reasoning. Furthermore, the finding that increased drinking times led to decreased DDV suggests the validity of this explanation. However, further detailed assessments, including conscious animal blood collection and precise urine production measurements, are necessary to identify the potential water diuresis mechanism underlying the beneficial effects of PDV restriction.

The current strategies for preventing congestion are primarily dependent on pharmacological diuretics, such as those involving loop diuretics and AVP antagonists (Houston et al. 2015). Such pharmaceutical agents act directly on the end organs and, because there is no feedback regulation mechanism, the volume is forcibly unloaded, increasing sympathetic activity and inducing renal AVP receptor expression (Francis et al. 1985; Bayliss et al. 1987; Felker et al. 2009). Despite the acute effectiveness of these agents in volume unloading and in the alleviation of CHF symptoms, their long‐term performance is not as good as expected(Konstam et al. 2007; Felker et al. 2009; Wu et al. 2014; Ogawa et al. 2015). Restricting daily intake volumes to prevent fluid retention may appear intuitively effective; however, this method may lead to inadequate fluid intake and neglects the powerful physiological stimulus that drinking provides for suppressing AVP secretion, leading to water diuresis.

In this study, we showed that frequently drinking small volumes exerted benefits for CHF rats, which may be partly mediated through the water diuresis mechanism. In fact, cardiologists commonly use ice chips to relieve thirst in patients with CHF (Allida et al. 2016). Unfortunately, even if cold water is known to promptly decrease vasopressin levels in normal persons, no studies have been performed in this field, to date (Salata et al. 1987).

Considering the limitations of this study, we recognize that the present CHF model is consistent with the previously reported large MI rodent model. Litwin et al. (1994) reported that the left ventricular (LV) end diastolic diameter (LVEDD), 6 weeks after MI induction in male Sprague‐Dawley rats, was 10.1 ± 0.9 mm. In our study, the LVEDD, measured 7 weeks after MI induction in the FD group was 11.4 ± 0.8 mm. The postmortem ventricle weights were similar (Litwin et al. reported 3.22 g/kg vs. our 3.28–3.43 g/kg). Furthermore, our pathological examinations showed that most of the LV anterior wall became scarred (MI size, approximately 50% at death). Therefore, the present model of CHF differs from human MI pathology as the rapid evolution of necrosis and cardiac dilation occurred during the first several weeks after MI induction. When interpreting data from a basic study, the differences between the animal model and human pathology must be recognized.

In previous studies, we proposed novel strategies for restoring imbalanced autonomic function using pharmacological or nonpharmacological methods in an animal model of CHF (Li et al. 2004, 2014). Although the mechanisms remain unclear, stimulation of the vagal afferent nerve is known to suppress AVP secretion, which reportedly plays a role in the long‐term benefits (Li et al. 2005). We believe that these novel approaches may involve a central mechanism that restores neurohumoral balance in CHF. In theory, these novel strategies differ from the current drug therapy, which relies on symptomatic treatment and direct targeting of peripheral organs like the heart and kidneys (e.g., beta‐blockers, diuretics). Although the acute effects of relieving symptoms through the central mechanism may not be as strong as those mediated by drugs acting on peripheral organs, the effects are mediated by the physiological regulation mechanism and are more likely to yield long‐term benefits. Nevertheless, the potential benefits of these novel strategies include this study proposed drinking behavior modulation need to be verified in future clinical research studies.

## Conclusion

In summary, the increased spontaneous PDV may be an intrinsic response to compensatory sympathetic activation after MI. Furthermore, a large PDV was strongly correlated with a short lifespan in CHF rats. This implies that a large PDV may be an unreported risk factor in CHF. Thus, modulating drinking behavior to include frequent drinking of small volumes resulted in improved cardiac function and survival in CHF rats. These data provide experimental evidence for future clinical research studies investigating novel fluid management strategies for patients with CHF.

## Conflict of Interest

None declared.
